# *N*-[4-(Morpholinodiazen­yl)phen­yl]acetamide

**DOI:** 10.1107/S160053680904937X

**Published:** 2009-11-25

**Authors:** Taylor Chin, Frank R. Fronczek, Ralph Isovitsch

**Affiliations:** a Department of Chemistry, Whittier College, 13406 Philadelphia Street, Whittier, CA 90608, USA; b Department of Chemistry, Louisiana State University, Baton Rouge, LA 70803, USA

## Abstract

The title compound, C_12_H_16_N_4_O_2_, is a member of a family of morpholine-substituted aromatic diazenes. Conjugation of the diazene group π-system and the lone pair of electrons of the morpholine N atom is evidenced by a lengthened N=N double bond of 1.2707 (19) Å and a shortened N—N single bond of 1.346 (2) Å. The bond angles at the morpholine N atom range from 113.52 (14) to 121.12 (14)°, indicating some degree of *sp* ^2^ hybridization. The morpholine ring adopts a conventional chair conformation with the diazenyl group in the equatorial position. The diazenyl and acetamido groups are both twisted relative to the plane of the benzene ring by 12.3 (2) and 25.5 (3)°, respectively.

## Related literature

The title compound was synthesized using a modification of the method of Sengupta *et al.* (1998 ▶). For similar structures, see: Little *et al.* (2008 ▶). For information about diazene derivatives, see: Chen *et al.* (2005 ▶); Lalezari & Afgahi (1975 ▶). For bond-length data, see: Allen *et al.* (1987 ▶).
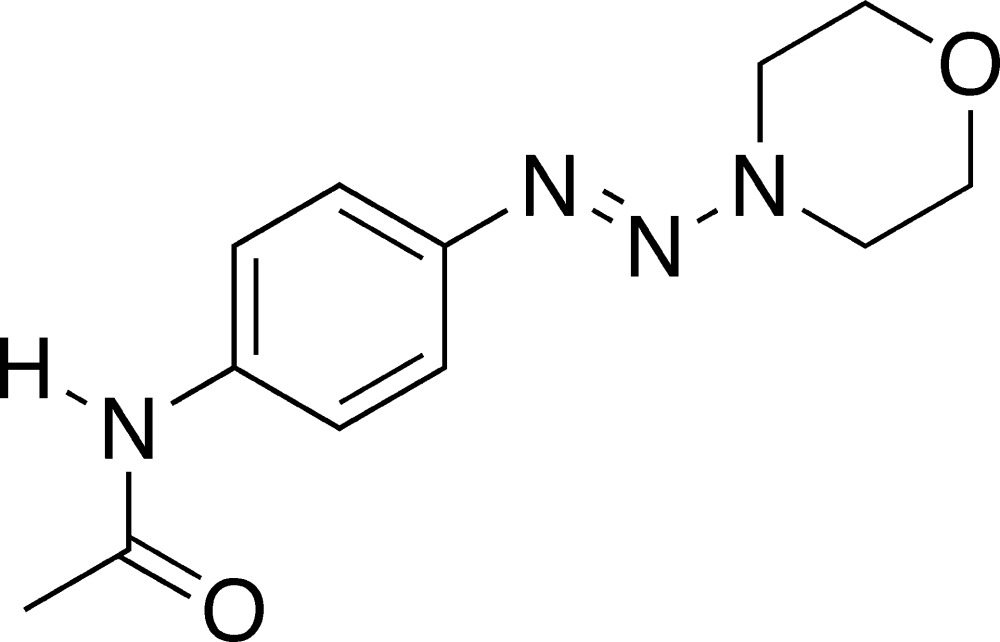


## Experimental

### 

#### Crystal data

C_12_H_16_N_4_O_2_
*M* *_r_* = 248.29
Monoclinic, 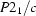

*a* = 12.6013 (4) Å
*b* = 10.6114 (3) Å
*c* = 9.2967 (2) Å
β = 93.874 (2)°
*V* = 1240.29 (6) Å^3^
*Z* = 4
Cu *K*α radiation
μ = 0.77 mm^−1^
*T* = 90 K
0.23 × 0.17 × 0.01 mm

#### Data collection

Bruker Kappa APEXII CCD area-detector diffractometer
Absorption correction: multi-scan (*SADABS*; Sheldrick, 2004 ▶) *T*_min_ = 0.843, *T*_max_ = 0.992
11437 measured reflections
2249 independent reflections
1655 reflections with *I* > 2σ(*I*)
*R*_int_ = 0.053

#### Refinement

*R*[*F* ^2^ > 2σ(*F* ^2^)] = 0.038
*wR*(*F* ^2^) = 0.095
*S* = 1.03
2249 reflections
168 parameters
H atoms treated by a mixture of independent and constrained refinement
Δρ_max_ = 0.20 e Å^−3^
Δρ_min_ = −0.20 e Å^−3^

### 

Data collection: *APEX2* (Bruker, 2006 ▶); cell refinement: *SAINT* (Bruker, 2006 ▶); data reduction: *SAINT*; program(s) used to solve structure: *SHELXS97* (Sheldrick, 2008 ▶); program(s) used to refine structure: *SHELXL97* (Sheldrick, 2008 ▶); molecular graphics: *ORTEP-3 for Windows* (Farrugia, 1997 ▶); software used to prepare material for publication: *SHELXTL* (Sheldrick, 2008 ▶).

## Supplementary Material

Crystal structure: contains datablocks I, global. DOI: 10.1107/S160053680904937X/gk2239sup1.cif

Structure factors: contains datablocks I. DOI: 10.1107/S160053680904937X/gk2239Isup2.hkl

Additional supplementary materials:  crystallographic information; 3D view; checkCIF report

## Figures and Tables

**Fig. 1.**
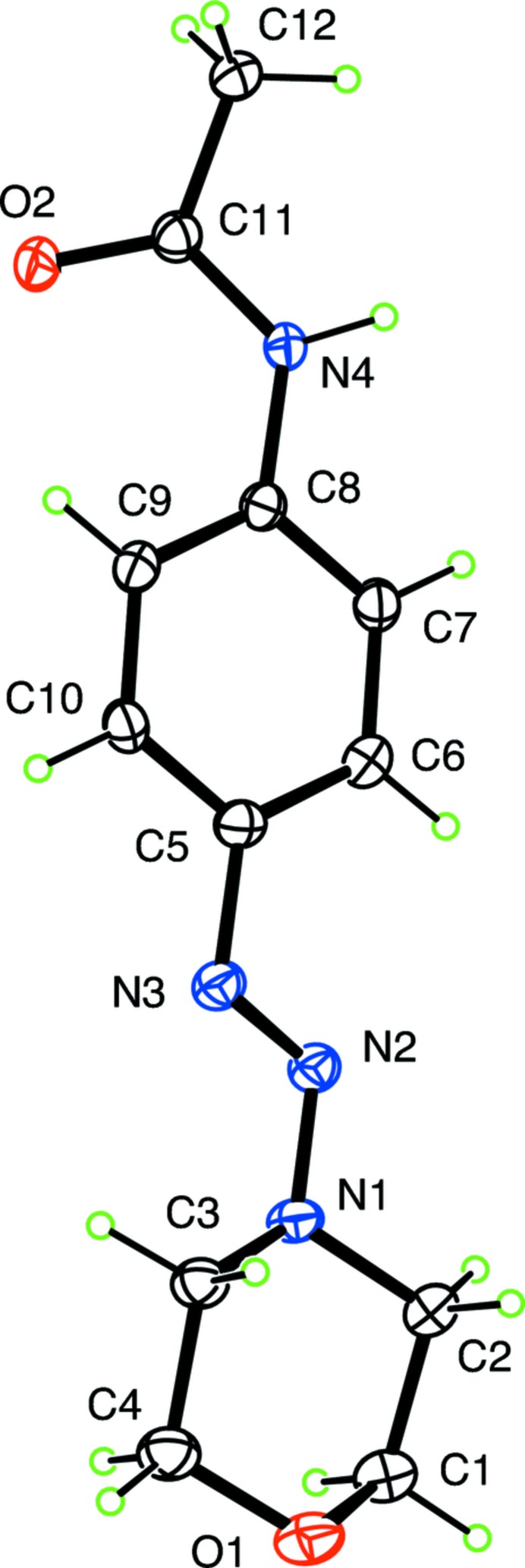
Molecular structure of the title compound with displacement ellipsoids at the 50% probability level. H atoms are shown with arbitrary radius.

## supplementary crystallographic information

### Comment

Diazene derivatives have found utility in various research areas (Lalezari & Afgahi, 1975; Chen *et al.*, 2005). Our research uses morpholine-substituted aryl diazenes as easily handled and prepared equivalents for the *in situ* generation of diazonium ions that are then used in the synthesis of novel derivatives of *trans*–stilbene *via* a Heck-type reaction (Sengupta *et al.*, 1998).

The structure of the title compound is shown in Figure 1. The N–N double bond adopted a *trans*-configuration. A N3–N2–N1 bond angle of 113.93 (14) ° deviates from the optimal trigonal planar geometry by approximately 6°. The diazene moiety, N3–N2–N1, exhibits π –delocalization, evidenced by N1–N2 and N2–N3 bond lengths of 1.346 (2) and 1.2707 (19) Å respectively. These values are between literature value of 1.222 Å for a N–N double bond and 1.420 Å for a N(*sp*^2^)–N(*sp*^3^) single bond (Allen *et al.*, 1987) Morpholine nitrogen bond angles that ranged from 113.52 (14)–121.12 (14)° indicated that the morpholine nitrogen had some degree of *sp*^2^ hybridization and participated in π –delocalization. The morpholine ring adopted a conventional chair conformation,with the diazenyl group in the equitorial postion on the morpholine nitrogen, N3. The acetamino and diazene groups were found to be twisted 25.5 (3)° and 12.3 (2)° respectively from the plane of the phenyl ring. The structure of the title compound is similar to the structure of related diazenes (Little *et al.*, 2008).

### Experimental

Synthetic procedures were carried out using standard techniques. Solvents and reagents were used as received. Melting points were determined in open capillaries and are uncorrected. ^1^H and ^13^C NMR spectra were recorded on a Jeol ECX 300 MHz spectrometer using TMS as the internal standard. The IR spectrum was recorded as a KBr disk on a JASCO 460 F T–IR.

4.26 g of *N*–(4-aminophenyl)acetamide (28.4 mmol) was added to 12.5 ml of 6 *M* HCl in an ice water bath and cooled to 0° C to yield a light pink precipitate. The solid was maintained at 0° C, and a solution of 2.08 g (30.09 mmol) of NaNO_2_ in 4.0 ml H_2_O was added dropwise with stirring over ten minutes; a dark green brown solution resulted. After stirring for twenty minutes, 2.70 ml morpholine (2.74 g, 31.42 mmol) was added dropwise in 10 minutes. Then saturated K_2_CO_3_ was added until pH of 8 was reached, and solution was stirred for ten minutes: a yellow brown suspension resulted. The tan solid was collected using vacuum filtration, washed well with water and dried in air. The crude product was recrystallized from a 1:3 benzene:cyclohexane mixture to give 3.55 g (50.4%) of 4-[(*E*)-(acetamidophenyl)diazenyl]-morpholine as a tan microcrystalline solid.

m.p. 448-449 K. IR (KBr) 3294, 3055, 2971, 1664, 1600 cm^-1. 1^H NMR (300 MHz, CD_3_CN): 2.03 (s, 3H), 3.67 (m, 4H), 3.77 (m, 4H), 7.33 (d, 2H), 7.51 (d, 2H), 8.33 (s, 1H). ^13^C NMR (75 MHz, DMSO–d*6*): 24.54, 48.33, 66.05, 119.89, 121.27, 138.28, 145.50, 168.75 p.p.m.. *R*_f_ = 0.61 (ethyl acetate)

### Refinement

H atoms on C were placed in idealized positions with C—H distances 0.95 - 0.99 Å and thereafter treated as riding. A torsional parameter was refined for the methyl group. The N—H hydrogen atom was placed from a difference map, and its coordinates were refined. *U*_iso_ for H were assigned as 1.2 times *U*_eq_ of the attached atoms (1.5 for methyl).

### Crystal data

**Table:**

C_12_H_16_N_4_O_2_	*F*(000) = 528
*M**_r_* = 248.29	*D*_x_ = 1.330 Mg m^−^^3^
Monoclinic, *P*2_1_/*c*	Cu *K*α radiation, λ = 1.54178 Å
Hall symbol: -P 2ybc	Cell parameters from 1544 reflections
*a* = 12.6013 (4) Å	θ = 3.5–67.6°
*b* = 10.6114 (3) Å	µ = 0.77 mm^−^^1^
*c* = 9.2967 (2) Å	*T* = 90 K
β = 93.874 (2)°	Plate, colorless
*V* = 1240.29 (6) Å^3^	0.23 × 0.17 × 0.01 mm
*Z* = 4	

### Data collection

**Table:**

Bruker Kappa APEXII CCD area-detector diffractometer	2249 independent reflections
Radiation source: fine-focus sealed tube	1655 reflections with *I* > 2σ(*I*)
graphite	*R*_int_ = 0.053
phi and ω scans	θ_max_ = 68.8°, θ_min_ = 3.5°
Absorption correction: multi-scan (*SADABS*; Sheldrick, 2004)	*h* = −15→14
*T*_min_ = 0.843, *T*_max_ = 0.992	*k* = −12→12
11437 measured reflections	*l* = −7→11

### Refinement

**Table:**

Refinement on *F*^2^	Secondary atom site location: difference Fourier map
Least-squares matrix: full	Hydrogen site location: inferred from neighbouring sites
*R*[*F*^2^ > 2σ(*F*^2^)] = 0.038	H atoms treated by a mixture of independent and constrained refinement
*wR*(*F*^2^) = 0.095	*w* = 1/[σ^2^(*F*_o_^2^) + (0.0421*P*)^2^ + 0.3*P*] where *P* = (*F*_o_^2^ + 2*F*_c_^2^)/3
*S* = 1.03	(Δ/σ)_max_ < 0.001
2249 reflections	Δρ_max_ = 0.20 e Å^−^^3^
168 parameters	Δρ_min_ = −0.20 e Å^−^^3^
0 restraints	Extinction correction: *SHELXL97* (Sheldrick, 2008), Fc^*^=kFc[1+0.001xFc^2^λ^3^/sin(2θ)]^-1/4^
Primary atom site location: structure-invariant direct methods	Extinction coefficient: 0.0012 (2)

### Special details

**Table:**

Geometry. All e.s.d.'s (except the e.s.d. in the dihedral angle between two l.s. planes) are estimated using the full covariance matrix. The cell e.s.d.'s are taken into account individually in the estimation of e.s.d.'s in distances, angles and torsion angles; correlations between e.s.d.'s in cell parameters are only used when they are defined by crystal symmetry. An approximate (isotropic) treatment of cell e.s.d.'s is used for estimating e.s.d.'s involving l.s. planes.
Refinement. Refinement of *F*^2^ against ALL reflections. The weighted *R*-factor *wR* and goodness of fit *S* are based on *F*^2^, conventional *R*-factors *R* are based on *F*, with *F* set to zero for negative *F*^2^. The threshold expression of *F*^2^ > σ(*F*^2^) is used only for calculating *R*-factors(gt) *etc*. and is not relevant to the choice of reflections for refinement. *R*-factors based on *F*^2^ are statistically about twice as large as those based on *F*, and *R*- factors based on ALL data will be even larger.

### Fractional atomic coordinates and isotropic or equivalent isotropic displacement parameters (Å^2^)

**Table:**

	*x*	*y*	*z*	*U*_iso_*/*U*_eq_	
O1	0.58539 (10)	0.71775 (12)	0.68342 (13)	0.0281 (3)	
O2	0.91392 (10)	−0.19398 (12)	0.26306 (12)	0.0260 (3)	
N1	0.63437 (11)	0.46590 (13)	0.62337 (14)	0.0203 (3)	
N2	0.66849 (11)	0.34581 (13)	0.61964 (14)	0.0198 (3)	
N3	0.73809 (11)	0.32649 (13)	0.52939 (14)	0.0204 (3)	
N4	0.88774 (11)	−0.17257 (13)	0.50181 (15)	0.0179 (3)	
H4N	0.8936 (14)	−0.2115 (18)	0.5884 (18)	0.021*	
C1	0.52108 (14)	0.61813 (17)	0.73287 (19)	0.0249 (4)	
H1A	0.4609	0.6025	0.6610	0.030*	
H1B	0.4914	0.6434	0.8244	0.030*	
C2	0.58485 (14)	0.49823 (17)	0.75637 (18)	0.0224 (4)	
H2A	0.6404	0.5104	0.8357	0.027*	
H2B	0.5376	0.4289	0.7836	0.027*	
C3	0.69258 (14)	0.56752 (16)	0.55717 (18)	0.0218 (4)	
H3A	0.7116	0.5419	0.4598	0.026*	
H3B	0.7591	0.5859	0.6163	0.026*	
C4	0.62278 (15)	0.68384 (17)	0.54677 (19)	0.0258 (4)	
H4A	0.6637	0.7550	0.5095	0.031*	
H4B	0.5611	0.6679	0.4775	0.031*	
C5	0.77020 (13)	0.19715 (16)	0.52560 (17)	0.0181 (4)	
C6	0.74615 (13)	0.10620 (16)	0.62701 (17)	0.0199 (4)	
H6	0.7041	0.1279	0.7044	0.024*	
C7	0.78367 (13)	−0.01525 (16)	0.61454 (17)	0.0192 (4)	
H7	0.7660	−0.0773	0.6826	0.023*	
C8	0.84737 (13)	−0.04798 (16)	0.50304 (16)	0.0169 (4)	
C9	0.87227 (14)	0.04281 (16)	0.40320 (17)	0.0188 (4)	
H9	0.9155	0.0216	0.3270	0.023*	
C10	0.83377 (13)	0.16458 (16)	0.41515 (17)	0.0186 (4)	
H10	0.8512	0.2265	0.3468	0.022*	
C11	0.92053 (13)	−0.23624 (16)	0.38778 (17)	0.0189 (4)	
C12	0.96655 (14)	−0.36422 (16)	0.42131 (18)	0.0217 (4)	
H12A	0.9306	−0.4270	0.3579	0.033*	
H12B	0.9564	−0.3855	0.5220	0.033*	
H12C	1.0427	−0.3637	0.4058	0.033*	

### Atomic displacement parameters (Å^2^)

**Table:**

	*U*^11^	*U*^22^	*U*^33^	*U*^12^	*U*^13^	*U*^23^
O1	0.0306 (7)	0.0194 (7)	0.0348 (7)	−0.0004 (6)	0.0055 (6)	−0.0053 (6)
O2	0.0442 (8)	0.0191 (7)	0.0147 (6)	0.0039 (6)	0.0025 (5)	−0.0009 (5)
N1	0.0226 (7)	0.0150 (7)	0.0236 (7)	0.0021 (6)	0.0038 (6)	−0.0025 (6)
N2	0.0215 (7)	0.0184 (8)	0.0195 (7)	0.0007 (6)	0.0002 (6)	−0.0021 (6)
N3	0.0221 (7)	0.0192 (8)	0.0199 (7)	0.0012 (6)	0.0009 (6)	−0.0028 (6)
N4	0.0243 (8)	0.0160 (7)	0.0136 (7)	0.0016 (6)	0.0025 (6)	0.0012 (6)
C1	0.0229 (9)	0.0227 (10)	0.0293 (9)	0.0014 (8)	0.0027 (8)	−0.0037 (8)
C2	0.0232 (9)	0.0229 (10)	0.0215 (9)	0.0006 (7)	0.0040 (7)	−0.0033 (8)
C3	0.0244 (9)	0.0168 (9)	0.0247 (9)	−0.0009 (8)	0.0042 (8)	−0.0008 (7)
C4	0.0296 (10)	0.0185 (9)	0.0294 (10)	−0.0005 (8)	0.0023 (8)	−0.0016 (8)
C5	0.0173 (8)	0.0173 (9)	0.0190 (8)	0.0002 (7)	−0.0025 (7)	−0.0032 (7)
C6	0.0197 (9)	0.0225 (10)	0.0179 (8)	−0.0005 (7)	0.0029 (7)	−0.0027 (7)
C7	0.0218 (9)	0.0198 (9)	0.0162 (8)	−0.0010 (7)	0.0016 (7)	0.0010 (7)
C8	0.0185 (8)	0.0169 (9)	0.0148 (8)	−0.0001 (7)	−0.0018 (7)	−0.0022 (7)
C9	0.0214 (8)	0.0198 (9)	0.0152 (8)	0.0001 (7)	0.0018 (7)	−0.0013 (7)
C10	0.0220 (8)	0.0180 (9)	0.0157 (8)	−0.0021 (7)	0.0001 (7)	0.0023 (7)
C11	0.0207 (9)	0.0180 (9)	0.0179 (9)	−0.0009 (7)	0.0010 (7)	−0.0009 (7)
C12	0.0277 (9)	0.0182 (9)	0.0194 (8)	0.0023 (8)	0.0030 (7)	−0.0020 (8)

### Geometric parameters (Å, °)

**Table:**

O1—C1	1.427 (2)	C3—H3B	0.9900
O1—C4	1.430 (2)	C4—H4A	0.9900
O2—C11	1.2407 (19)	C4—H4B	0.9900
N1—N2	1.346 (2)	C5—C10	1.388 (2)
N1—C2	1.463 (2)	C5—C6	1.397 (2)
N1—C3	1.463 (2)	C6—C7	1.380 (2)
N2—N3	1.2707 (19)	C6—H6	0.9500
N3—C5	1.432 (2)	C7—C8	1.397 (2)
N4—C11	1.345 (2)	C7—H7	0.9500
N4—C8	1.417 (2)	C8—C9	1.388 (2)
N4—H4N	0.903 (17)	C9—C10	1.387 (2)
C1—C2	1.513 (2)	C9—H9	0.9500
C1—H1A	0.9900	C10—H10	0.9500
C1—H1B	0.9900	C11—C12	1.501 (2)
C2—H2A	0.9900	C12—H12A	0.9800
C2—H2B	0.9900	C12—H12B	0.9800
C3—C4	1.515 (2)	C12—H12C	0.9800
C3—H3A	0.9900		
			
C1—O1—C4	109.15 (13)	O1—C4—H4B	109.2
N2—N1—C2	113.52 (14)	C3—C4—H4B	109.2
N2—N1—C3	121.12 (14)	H4A—C4—H4B	107.9
C2—N1—C3	115.93 (14)	C10—C5—C6	119.32 (16)
N3—N2—N1	113.93 (14)	C10—C5—N3	115.78 (15)
N2—N3—C5	112.31 (14)	C6—C5—N3	124.83 (14)
C11—N4—C8	127.19 (14)	C7—C6—C5	119.82 (15)
C11—N4—H4N	117.5 (12)	C7—C6—H6	120.1
C8—N4—H4N	115.3 (12)	C5—C6—H6	120.1
O1—C1—C2	111.22 (14)	C6—C7—C8	120.77 (16)
O1—C1—H1A	109.4	C6—C7—H7	119.6
C2—C1—H1A	109.4	C8—C7—H7	119.6
O1—C1—H1B	109.4	C9—C8—C7	119.38 (16)
C2—C1—H1B	109.4	C9—C8—N4	123.00 (15)
H1A—C1—H1B	108.0	C7—C8—N4	117.55 (14)
N1—C2—C1	109.14 (14)	C10—C9—C8	119.79 (15)
N1—C2—H2A	109.9	C10—C9—H9	120.1
C1—C2—H2A	109.9	C8—C9—H9	120.1
N1—C2—H2B	109.9	C9—C10—C5	120.90 (16)
C1—C2—H2B	109.9	C9—C10—H10	119.5
H2A—C2—H2B	108.3	C5—C10—H10	119.5
N1—C3—C4	108.78 (14)	O2—C11—N4	123.37 (15)
N1—C3—H3A	109.9	O2—C11—C12	121.44 (15)
C4—C3—H3A	109.9	N4—C11—C12	115.19 (14)
N1—C3—H3B	109.9	C11—C12—H12A	109.5
C4—C3—H3B	109.9	C11—C12—H12B	109.5
H3A—C3—H3B	108.3	H12A—C12—H12B	109.5
O1—C4—C3	111.89 (14)	C11—C12—H12C	109.5
O1—C4—H4A	109.2	H12A—C12—H12C	109.5
C3—C4—H4A	109.2	H12B—C12—H12C	109.5
			
C2—N1—N2—N3	−159.87 (14)	N3—C5—C6—C7	−178.36 (16)
C3—N1—N2—N3	−14.8 (2)	C5—C6—C7—C8	1.3 (3)
N1—N2—N3—C5	−178.35 (13)	C6—C7—C8—C9	−0.5 (2)
C4—O1—C1—C2	62.57 (18)	C6—C7—C8—N4	176.59 (16)
N2—N1—C2—C1	−163.16 (14)	C11—N4—C8—C9	−25.5 (3)
C3—N1—C2—C1	49.87 (19)	C11—N4—C8—C7	157.45 (16)
O1—C1—C2—N1	−55.31 (19)	C7—C8—C9—C10	0.0 (2)
N2—N1—C3—C4	166.58 (14)	N4—C8—C9—C10	−176.97 (16)
C2—N1—C3—C4	−49.15 (19)	C8—C9—C10—C5	−0.2 (3)
C1—O1—C4—C3	−62.36 (19)	C6—C5—C10—C9	0.9 (2)
N1—C3—C4—O1	54.28 (19)	N3—C5—C10—C9	178.10 (15)
N2—N3—C5—C10	170.70 (14)	C8—N4—C11—O2	−4.0 (3)
N2—N3—C5—C6	−12.3 (2)	C8—N4—C11—C12	175.96 (16)
C10—C5—C6—C7	−1.4 (2)		

### Hydrogen-bond geometry (Å, °)

**Table:**

*D*—H···*A*	*D*—H	H···*A*	*D*···*A*	*D*—H···*A*
N4—H4N···O2^i^	0.903 (17)	1.911 (18)	2.8115 (18)	174.5 (17)

Symmetry codes: (i) *x*, −*y*−1/2, *z*+1/2.
